# Mitochondrial Contact Sites in Inflammation-Induced Cardiovascular Disease

**DOI:** 10.3389/fcell.2020.00692

**Published:** 2020-07-30

**Authors:** Hao Liu, Xiao Liu, Haixia Zhuang, Hualin Fan, Dongxing Zhu, Yiming Xu, Pengcheng He, Jinbao Liu, Du Feng

**Affiliations:** ^1^Affiliated Cancer Hospital & Institute of Guangzhou Medical University, Guangzhou, China; ^2^Guangzhou Municipal and Guangdong Provincial Key Laboratory of Protein Modification and Degradation, State Key Laboratory of Respiratory Disease, School of Basic Medical Sciences, Guangzhou Medical University, Guangzhou, China; ^3^Guangdong Provincial People’s Hospital, School of Medicine, South China University of Technology, Guangzhou, China; ^4^Guangzhou Institute of Cardiovascular Diseases, The Second Affiliated Hospital, Key Laboratory of Cardiovascular Diseases, School of Basic Medical Sciences, Guangzhou Medical University, Guangzhou, China; ^5^School of Basic Medical Sciences, Guangzhou Medical University, Guangzhou, China; ^6^Department of Cardiology, Guangdong Cardiovascular Institute, Guangdong Provincial Key Laboratory of Coronary Heart Disease Prevention, Guangdong Provincial People’s Hospital, Guangdong Academy of Medical Sciences, Guangzhou, China

**Keywords:** mitochondrial-associated membranes, mitochondria, autophagy, cardiovascular disease, inflammation, inflammasome

## Abstract

The mitochondrion, the ATP-producing center, is both physically and functionally associated with almost all other organelles in the cell. Mitochondrial-associated membranes (MAMs) are involved in a variety of biological processes, such as lipid exchange, protein transport, mitochondrial fission, mitophagy, and inflammation. Several inflammation-related diseases in the cardiovascular system involve several intracellular events including mitochondrial dysfunction as well as disruption of MAMs. Therefore, an in-depth exploration of the function of MAMs will be of great significance for us to understand the initiation, progression, and clinical complications of cardiovascular disease (CVD). In this review, we summarize the recent advances in our knowledge of MAM regulation and function in CVD-related cells. We discuss the potential roles of MAMs in activating inflammation to influence the development of CVD.

## Introduction

Inflammation is a universal phenomenon observed in CVDs, such as atherosclerosis (the primary underlying cause), AMI, cardiac I/R injury, stroke, and HF (Golia et al., 2014; Esposito et al., 2017; Zhou et al., 2018). Many experimental and clinical studies suggest that treatment with anti-inflammatory drugs is capable of reducing the risk of CVDs (Golia et al., 2014; Maffia and Cirino, 2017; Ridker et al., 2017). Generally, inflammation is classified into classic inflammation (which is caused by infection and tissue injury) and para-inflammation (which is caused by tissue stress or malfunction); the latter is more responsible for chronic inflammatory disease, including T2D and CVDs (Medzhitov, 2008). The inflammasome, a critical factor in pro-inflammation, participates in a variety of inflammatory diseases and also serves as a possible therapeutic target for infectious and inflammatory diseases (Rathinam and Fitzgerald, 2016). In activated macrophages, NLRP3 is situated downstream of a series of signaling events including generation and releasing of mtROS (Tschopp and Schroder, 2010), cytosolic mtDNA release (Nakahira et al., 2011), lysosomal damage (Hornung and Latz, 2010), and cytosolic K^+^ efflux (Petrilli et al., 2007; Shimada et al., 2012). Recent research has suggested that MAMs are crucial platforms for inflammasome formation. Impaired Ca^2+^ flux between mitochondria and the ER causes mitochondrial damage that, in turn, induces the activation of the NLRP3 inflammasome (Missiroli et al., 2018). In addition, the NLRP3 inflammasome is recruited to MAMs and activated by MAM-related effectors (Zhang et al., 2017). Given the importance of MAMs in the pathogenesis of CVDs, we hereby summarize the progress in understanding the specific roles of MAMs in inflammasome activation and the association between MAMs and the high-risk factors of CVDs. We also highlight some new research ideas in inflammasome-induced CVDs.

## MAMs, Inflammasomes, and CVDs

About 2.5 billion years ago, a bacterium that used oxygen to convert organic molecules into energy turned into a mitochondrion after it was engulfed by an archaebacterium (McInerney et al., 2015). Eventually, it evolved into a double-membrane organelle inside eukaryotic cells, and provided energy for the cell while also participating in other cellular biological functions (Mills et al., 2017). This is a hypothesis of the origination of mitochondria which have been identified as the stable structures in cells and actively participate in cellular metabolism.

The organelles, including mitochondria and the ER in eukaryotic cells, are isolated from each other by their membranes, which allow individual organelles to have independent microenvironments to facilitate the appropriate biochemical reactions. Organelles are also tightly connected and work in a coordinated manner. When cells perform biological functions, some of the organelles need to be close to each other (Porter and Palade, 1957). In Bernhard and Rouiller (1956) first discovered Membrane Contact Sites (MCSs) with ultrastructural studies (Gatta and Levine, 2017). In 1969, John Ruby and his colleagues found a possible interaction between the outer mitochondrial membrane and the ER membrane (van Vliet et al., 2014). Shore and Tata used the same approach in rat liver homogenates with low-speed (640 g) to extract the rough and smooth ER fractions and found the major proportion of mitochondria was in this fractions at the end of the 1970s (Shore and Tata, 1977a, b). It was not until the last decade that the functions of MCSs have been gradually revealed (Gatta and Levine, 2017). MCSs are formed by interconnecting membrane protein complexes and lipids, which keep the two organelles in close contact without fusion (Annunziata et al., 2018). As one type of MCS, the MAM is the membrane contact between mitochondria and the ER, and it plays a role in exchange of materials and transport of ions between these organelles (Rizzuto et al., 1993). In recent years, MAMs have been found to be involved in intracellular phospholipid transport (Vance, 1990; Friedman et al., 2018), mitophagy (Missiroli et al., 2016; Wu et al., 2016), energy metabolism (Csordas and Hajnoczky, 2009), mitochondrial morphology (Liu et al., 2009), apoptosis (Theurey and Rieusset, 2017), and inflammasome formation (Zhou et al., 2011). These biological functions seem to be independent of each other, but they are inseparably linked. The MAM maintains the physiological function of normal cells within tissues, and the imbalance of MAMs is implicated in various diseases (Simmen and Tagaya, 2017; Wong et al., 2019). The disturbance of MAMs will lead to abnormal intracellular Ca^2+^ levels, impaired lipid transport, and the destruction of mitochondria. Consequently, the dysfunction of MAMs is associated with various diseases, such as cancers (Peretti et al., 2019), neurodegeneration (Krols et al., 2016), diabetes (Rutter and Pinton, 2014), infection (Jacobs and Coyne, 2013), and CVDs (Eisner et al., 2013).

**FIGURE 1:**
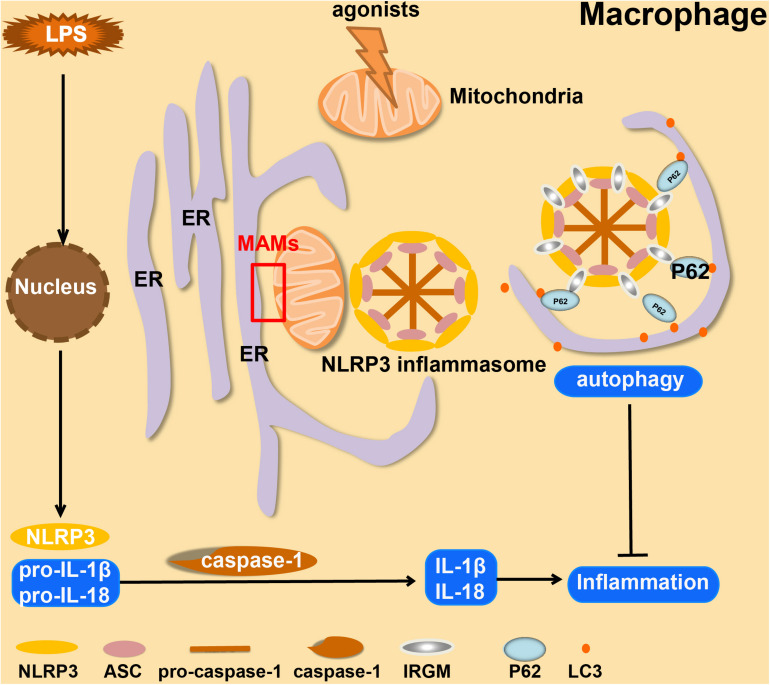
Overview of the formation and autophagic clearance of inflammasomes. MAMs form in macrophages when they are stimulated by LPS and exposed to NLRP3 agonists (such as ATP, Alum, and Nigericin). Under these conditions, mitochondria usually become unstable, and then ASC and NLRP3 will be recruited at the MAM, which forms a positive-feedback loop. In Crohn’s Disease, serving as a compensatory mechanism, IRGM promotes selective autophagy to suppress the formation of NLRP3 inflammasomes to limit inflammation.

In macrophages, the inflammasome transduces signals sensed by specific cytosolic proteins of the NLRP family into proteolytic activation of caspase-1 and -11, which can stimulate cells to yield and secrete cytokines including IL-1β, -18, and -1α (He et al., 2016; Yabal et al., 2019). During this process, there will be an increased level of mtROS and mtDNA released from mitochondria into the cytoplasm (Shimada et al., 2012). The increase of mtROS will recruit NLRP3 and cardiolipin to the outer membrane of mitochondria, as well as promoting K^+^ efflux from mitochondria. Subsequently, ASC (apoptosis-related speck-like protein) accumulates at MAMs where the NLRP3-ASC complex is formed, stimulating caspase-1 activation (Zhou et al., 2011; Elliott et al., 2018; Namgaladze et al., 2019). These events lead to activation of macrophages. Due to changes in mitochondrial morphology and function, acetylation of tubulin occurs, which in turn increases the abundance of MAMs (Yabal et al., 2019; Figure 1). However, there are still many unexplored steps in this complex pathway. The molecular mechanisms that initiate the functional and morphological changes in mitochondria in activated macrophages have not yet been elucidated. These are, therefore, future research directions in this field.

## Atherosclerosis

**FIGURE 2:**
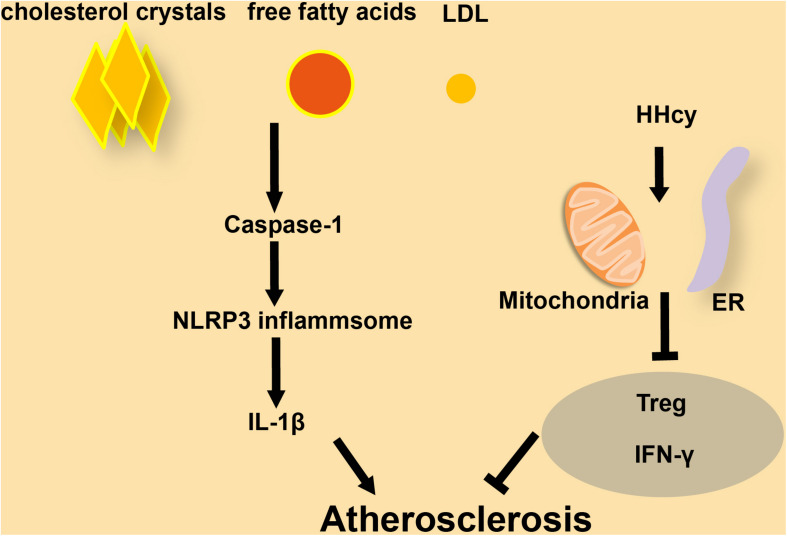
The role of inflammasomes and MAMs in atherosclerosis. **(A)** In macrophages, cholesterol crystals, free fatty acids, and LDL increase the progression of atherosclerosis by activating caspase-1 and NLRP3 inflammasome formation, followed by release of secreted factors. **(B)** In T-cells, HHcy increases ER-mitochondria coupling, which attenuates IFN-γ secretion and suppresses Treg cells to accelerate atherosclerosis.

Cardiovascular diseases, including CAD, cardiomyopathy, and HF, are associated with a high incidence of mortality (Mathers and Loncar, 2006; North and Sinclair, 2012). Atherosclerosis is a chronic disease that leads to progressive stenosis of arteries due to an imbalance in lipid metabolism (Guo et al., 2015). Immunocytes and cholesterol crystals accumulate on the arterial wall, leading to the development of an atherosclerotic plaque, which limits the flow of blood, and therefore of nutrients and oxygen, to various organs (Weber and Noels, 2011). Atherosclerosis can lead to further complications including life-threatening CVDs such as myocardial infarction and stroke (Sing et al., 2003; Guo et al., 2015). Atherosclerosis is more likely to occur in the colon germ-free animals, which suggests that atherosclerosis is closely related to inflammation caused by endogenous substances (Wright et al., 2000). Prevention and treatment of early atherosclerosis will deliver a breakthrough in the treatment of CVDs. NLRP3 is involved in the sterile inflammatory response in a variety of disease conditions (Liu et al., 2018). There are tiny cholesterol crystals in early atherosclerotic lesions. These crystals cause inflammation through activated caspase-1 which is cleaved by NLRP3 inflammasomes. The latter will lead to the secretion of cytokines like the IL family, which in turn will induce the formation and development of atherosclerotic plaques (Duewell et al., 2010). As the first signal for inflammasome activation, the abnormal accumulation of free fatty acids and LDL in human blood caused by imbalanced lipid metabolism can promote the production of pro-IL-1β through Toll-like receptors (Masters et al., 2011; Figure 2). Mice without LDL receptors are prone to develop atherosclerotic plaques. Compared to wild-type mice, atherosclerotic lesions were significantly reduced in NLRP3- or ASC- knockout mice after feeding them a high-cholesterol diet (Duewell et al., 2010). Similarly, the atherosclerotic lesions will shrink after depletion of IL-1β in ApoE-deficient mice (Kirii et al., 2003; Bhaskar et al., 2011). PACS-2 (phosphofurin acidic cluster sorting protein 2) regulates the distance between ER and mitochondria. Reduced expression of PACS-2 induces uncoupling of mitochondria from the ER and BAP31-dependent mitochondrial fission (Simmen et al., 2005). Upon stimulation with atherogenic lipids, PACS-2-associated MAM contacts increase in human VSMCs (Moulis et al., 2019). In VSMCs lacking PACS-2, MAM formation is impaired, leading to reduced mitophagosome formation and increased apoptosis induced by oxidized lipoprotein (Moulis et al., 2019). HHcy has been identified as a high-risk factor for CVDs from a mass of clinical studies. In T-cells, Hcy increases the association of mitochondria with the ER. However, Nocodazole enlarges the distance between ER and mitochondria, leading to the inhibition of IFN-γ secretion and proliferation of T-cells. This effect shows that the balance of MAMs is essential for T-cell activation (Feng et al., 2016). Hcy accelerates atherosclerosis by increasing the release of chemokines/cytokines in monocytes and T-cells and results in the dysfunction of regulatory T-cells (Zeng et al., 2003; Feng et al., 2009; Ma et al., 2013; Figure 2). In macrophages from patients with CAD, the Ca^2+^ flux through MAMs maintains mitochondrial hyperactivity when GSK3b is inactivated, leading to the production of the collagenase cathepsin K that is related to CAD (Zeisbrich et al., 2018). According to existing research mentioned above (Moulis et al., 2019), the abundance of MAMs significantly increases in VSMCs and a subset of immune cells in atherosclerosis. At the same time, the increased MAMs in these immune cells will promote the release of inflammatory factors and further aggravate the development of atherosclerosis.

## Cardiomyopathy

**FIGURE 3:**
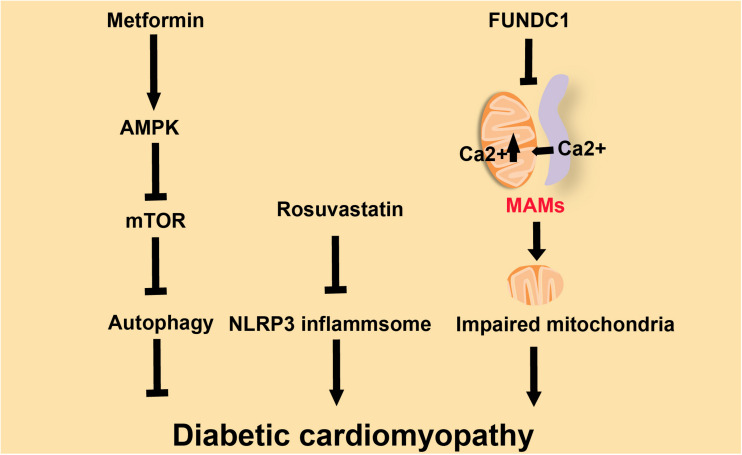
Three factors influence diabetic cardiomyopathy. Metformin ameliorates diabetic cardiomyopathy via activation of AMPK, which inhibits mTOR and promotes autophagy. Rosuvastatin improves diabetic cardiomyopathy by repressing NLRP3-inflammasomes. FUNDC1 down-regulates Ca^2+^ flux and subsequently improves diabetic cardiomyopathy.

Cardiomyopathy is defined as a myocardial disorder in which the heart muscle is functionally and structurally abnormal (McKenna et al., 2017). There are several different types of cardiomyopathies, and they can either be acquired, such as diabetic cardiomyopathy, or inherited, such as familial DCM (McKenna et al., 2017; Schaufelberger, 2019). Diabetic cardiomyopathy, one of the complications caused by diabetes, is closely related to the increased incidence of HF and arrhythmia in diabetic individuals (Boudina and Abel, 2007). Downregulation of the NLRP3 inflammasome restores cardiac function in diabetic cardiomyopathy models (Li et al., 2014; Yang et al., 2018; Figure 3). Metformin, the most widely used drug for treating T2D (Forslund et al., 2017), can inhibit NLRP3 by activating AMPK (AMP-activated protein kinase), thus increasing autophagy activity to promote the clearance of inflammasomes via inhibiting the mTOR pathway and alleviating the symptoms in diabetic cardiomyopathy (Yang et al., 2019a; Figure 3). Rosuvastatin can effectively delay the progress of diabetic cardiomyopathy through inhibition of NLRP3 inflammasomes (Figure 3; Luo et al., 2014). Familial DCM, a syndrome caused by genetic mutation, is characterized by an enlarged heart and impaired contractile function (Hershberger and Siegfried, 2011; Caragnano et al., 2019). DCM hearts are marked by the accumulation of lipoperoxidation products and the activation of both inflammasome and redox-responsive pathways (Caragnano et al., 2019). In mice, absence of the protein FUNDC1 (FUN14 domain containing 1) impaires the structure of MAMs, leads to the fusion of mitochondria and causes mitochondrial dysfunction, which results in DCM. In wild-type mice, MAM-localized FUNDC1 maintains the Ca^2+^ balance between mitochondria and the cytosol by influencing the function of IP3R2 (inositol 1,4,5-trisphosphate type 2 receptor). The absence of FUNDC1 at MAMs leads to the fusion of mitochondria and causes mitochondrial dysfunction, which results in DCM (Wu et al., 2017). FUNDC1 can also mediate diabetes-induced MAM formation and mitochondrial Ca^2+^ increase, resulting in impairment of cardiac structure and function (Munoz and Zorzano, 2017; Figure 3). Diabetes induces MAM formation through the downregulation of AMPK, and eventually causes diabetic cardiomyopathy (Wu et al., 2019). The inflammasome is essential to the development of diabetic cardiomyopathy and DCM (Li et al., 2014; Yang et al., 2018; Caragnano et al., 2019). The loss of NLRP3 will reduce heart damage in cardiomyopathy. The stability of MAMs is important for the structure and function of the heart. For example, an imbalance of MAMs will increase the concentration of Ca^2+^ in mitochondria, thereby destroying the mitochondria. The abundance of MAMs may affect the internal microenvironment of cardiomyocytes, including the ion levels and mitochondria-related events, in cardiomyopathy.

## Heart Failure

**FIGURE 4:**
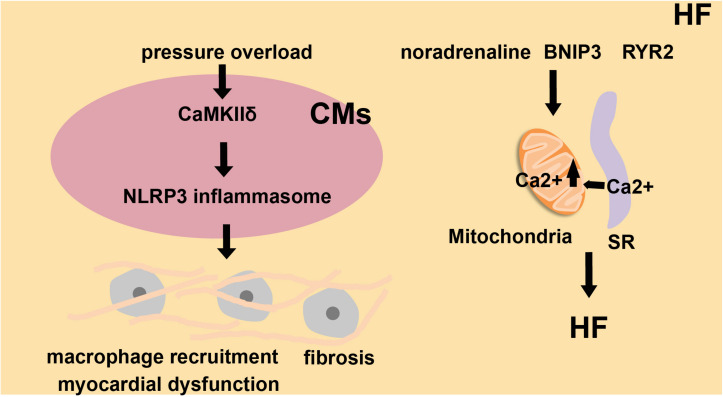
The role of inflammasomes and MAMs in heart failure (HF). Under high blood pressure, cardiomyocytes (CMs) activate CaMKIIδ, trigger inflammatory gene expression and activate NLRP3 inflammasomes. This leads to macrophage recruitment, fibrosis, and myocardial dysfunction, which finally induces heart failure. Noradrenaline, BNIP3 and RYR2 channels influence SR-to-mitochondria Ca^2+^ transfer and also alter cardiac metabolism. These factors all contribute to HF.

The prevalence of HF is closely related to aging and approximately doubles with each decade. Due to the increase of the aging population, the threat of HF to humans will gradually increase in the coming decades (Sano et al., 2018). HF is related to chronic sterile inflammation induced by the activation of the inflammasome, which produces inflammatory cytokines that accelerate the process of myocardial apoptosis and ultimately lead to cardiac hypertrophy (Butts et al., 2018). Mice with Tet2 (Ten-eleven translocation 2)- deficient hematopoietic cells show more maladaptive cardiac remodeling and dysfunction in two HF models (transverse aortic constriction and the permanent ligation of the left anterior descending artery). IL-1β blockade or administration of an NLRP3 inflammasome inhibitor provided effective protection in these models (Sano et al., 2018). CaMKIIδ (Ca^2+^/calmodulin-regulated kinase δ) will be activated in cardiomyocytes, followed by NLRP3 inflammasome activation (Figure 4). These responses promote macrophage recruitment, fibrosis, and HF induced by myocardial dysfunction (Suetomi et al., 2018). The SR, the ER in muscle cells, is associated with mitochondria, and this association is essential to the normal physiological functions of muscle cells (Dorn and Maack, 2013; Lopez-Crisosto et al., 2017). In noradrenaline-treated cardiomyocytes, cardiac metabolism is disordered due to the increased distance between the SR and mitochondria and the imbalanced Ca^2+^ homeostasis (Gutierrez et al., 2014). Overexpression of BNIP3 (BCL2/adenovirus E1B interacting protein 3) induces the oligomerization of VDAC1 (voltage-dependent anion-selective channel protein 1), which increases Ca^2+^ flux through MAMs into the mitochondria from SR. Based on this, BNIP3 induces mitochondrial dysfunction and apoptosis of cardiomyocytes, and eventually contributes to HF (Chaanine et al., 2013; Figure 4). In mice with leaky RYR2 (ryanodine receptor type 2) channels caused by genetic mutation, there is a detrimental increase in mitochondrial Ca^2+^ levels from the SR through MAMs. This Ca^2+^ leak also causes alterations of mitochondrial function and morphology (Santulli et al., 2015). Another report showed that Ca^2+^ released from the SR tunneled to mitochondria via RyRs, as IP3 receptors presented on mitochondrial and SR, leading to mitochondrial ATP production (Seidlmayer et al., 2016; Figure 4). MAMs are essential channels for Ca^2+^ to flow into the mitochondria from the SR. In cardiomyocytes, the excessive loading of Ca^2+^ into the mitochondria is a key contributor to mitochondrial imbalance, which in turn causes myocardial hypertrophy, and ultimately leads to HF.

## Autophagy and Inflammasome Clearance

Mitochondrial homeostasis is essential for heart health. Damaged mitochondria have reduced ATP production and yield dangerous amounts of ROS. Accumulated ROS may damage respiratory complex proteins, membrane lipids, and mtDNA, leading to catastrophic oxidative damage to the feed-forward cycle, and ultimately to cell death (Whelan et al., 2010; Morales et al., 2020). Damaged mitochondria, ubiquitinated in a Parkin-dependent manner, will be specifically recognized by p62, which induces autophagy (Zhong et al., 2016). The inflammasome components, ASC, NLRP3, and pro-caspase-1, accumulate at the MAMs (Zhou et al., 2011). In Crohn’s Disease, the expression of IRGM (immunity-related GTPase M) is up-regulated to promote the accumulation of p62 and LC-3 around the NLRP3 inflammasome, which is then selectively removed by autophagy (Mehto et al., 2019a, b). Through this process, macrophages clear the MAM-associated inflammasomes, which is a cellular “self-defense” response to inflammatory stresses (Moulis et al., 2019; Figure 1). It is generally believed that a controlled inflammatory response is beneficial (for example, to protect against infection), but it can be harmful if dysregulated (for example, causing septic shock). Regardless of the cause, inflammation is presumably an adaptive response to restore homeostasis (Medzhitov, 2008). The NF-κB pathway is an inflammatory signaling pathway. In macrophages, activation of the NF-κB signaling pathway inhibits the inflammatory activity through p62-induced elimination of damaged mitochondria (Zhong et al., 2016).

## Cardiovascular Drugs and MAM-Related CVDs

Several drugs are already in use to treat MAM-related CVDs. Nocodazole attenuates IFN-γ secretion and proliferation of T-cells (Feng et al., 2016) and reduces the inflammatory response by uncoupling MAMs. Consequently, it decreases the development of atherosclerotic plaques. Both Metformin (Yang et al., 2019a) and Rosuvastatin (Luo et al., 2014) can improve diabetic cardiomyopathy by inhibiting the formation of inflammasomes in diabetic patients. Noradrenaline, as an inducer of cardiac hypertrophy, alters Ca^2+^ handling and cardiac metabolism through MAMs (Gutierrez et al., 2014). Perhaps we could administer a norepinephrine inhibitor, such as Reboxetine, to improve the symptoms of HF patients. Recently, several clinical trials have reported that novel pharmacological therapies are associated with better outcomes in CVD patients. Canakinumab, an IL-1 antagonist, reduces the recurrence of ischemic events in patients with CVDs, and also reduces the hospitalization rate for HF (Abbate et al., 2020). Anakinra, a recombinant IL-1 receptor antagonist, has similar effects as canakinumab for CVD patients (Abbate et al., 2020). The influence of these therapies on the structure of MAMs in inflammation-induced CVDs should be further explored.

As described above, inflammation is a major factor in the occurrence and development of CVDs (Hansson and Hermansson, 2011), and cells need to repair themselves after exposure to inflammation. The formation and elimination of inflammasomes are completed at MAMs, which also participate in various biochemical functions such as Ca^2+^ communication and lipid transport. In summary, by studying the structure and function of MAMs, we may further understand the process of inflammasome formation and elimination. Interventions to inhibit the early inflammatory events would be beneficial to the treatment of CVD, and this will be a promising research direction in the CVD field in the future.

## MAMs and CVD Risk Factors

It has been shown that the abundance of inflammasomes increases in patients with a high risk of CVDs. The risk factors include obesity, smoking, diabetes, hypertension, and hypercholesterolemia. In the following sections, we will analyze the relationship between MAMs and these risk factors one by one.

## Obesity

**FIGURE 5:**
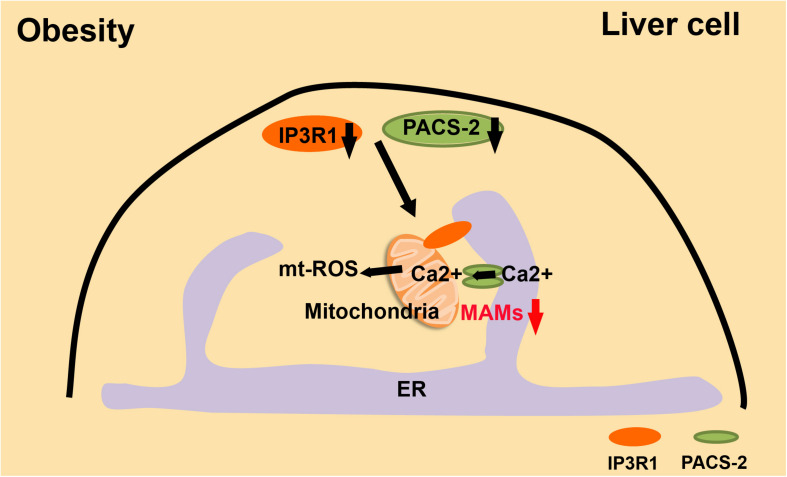
MAMs and the development of obesity. In obesity, liver cells have excessive MAMs, high mtROS, and Ca^2+^-overloaded mitochondria. These are all significantly relieved when IR3R1 and PACS2 are expressed at a lower level.

The balance between the immune system and metabolism is disturbed in obese individuals, which increases the risk of CVD (Hotamisligil, 2006). Overnutrition leads to dysfunction of membrane-bound organelles, such as ER and mitochondria (Lowell and Shulman, 2005; Hotamisligil, 2010). In obesity, mitochondrial ROS is increased and mitochondria are significantly overloaded with Ca^2+^. When the expression of IP3R1, the inositol triphosphate receptor, and PACS-2, the tethering protein of MAMs, is inhibited in obese mice, intracellular homeostasis is substantially improved, and obesity-induced metabolic imbalances are relieved (Arruda et al., 2014; Figure 5). The accumulation of MAMs is an early event in the process of obesity, and it is an adaptation process in the cell. However, long-term maintenance of MAMs will cause a series of mitochondrial dysfunctions, such as mitochondrial Ca^2+^ overload, reduced mitochondrial oxidative capacity, and increased mtROS.

In the pericentral zone of the liver, melatonin can convert macrosteatosis to microsteatosis. Melatonin increases the distance from ER to mitochondria, or decreases the abundance of MAMs, resulting in the improving of the structure and the metabolic functions of hepatic tissue (Stacchiotti et al., 2016). In summary, restoring the normal homeostasis of MAMs may reduce the degree of obesity and decrease the incidence of CVDs.

## Diabetes

**FIGURE 6:**
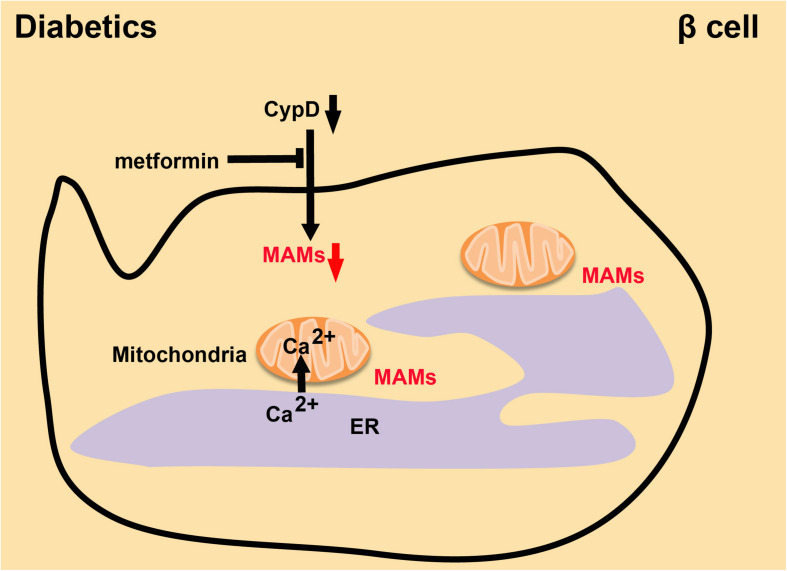
The integrity of MAMs in diabetes. Absence of CypD decreases the quantity of MAMs and damages their integrity, while Metformin reverses this effect and leads to recovery of insulin sensitivity.

The risk of CVDs in diabetes increases 2–4 fold (Faria and Persaud, 2017). Controlling blood glucose and enhancing insulin resistance will significantly decrease the risk of CVDs in diabetic individuals (Gilca et al., 2017). Serving as a crucial intracellular regulator in the function of insulin secretion by pancreatic beta cells, Ca^2+^ is kept at low intracellular levels in normal conditions. In a high-glucose environment, beta cells will close ATP-sensitive K^+^ channels and inhibit plasma membrane depolarization to facilitate the precise increase in cytoplasmic Ca^2+^ levels, leading to the secretion of insulin (Rutter et al., 2017). However, dysfunctional MAMs will lead to abnormal Ca^2+^ transport and imbalanced Ca^2+^ levels in the cell, resulting in the pathogenesis of T2D, which is caused by decreased insulin sensitivity (Wang and Wei, 2017). Palmitate increases chemokine production from the islets, which promotes immune cell infiltration into the islets and increases the levels of immunocyte in the islets of patients, thus inducing insulin resistance (Rieusset et al., 2012; Tubbs et al., 2014; Khodabandehloo et al., 2016). In HuH7 hepatocellular carcinoma cells, increased MAM formation prevents the alteration of insulin signal transduction induced by palmitate (Tubbs et al., 2014). The integrity of MAMs is necessary for insulin signal transduction. Deletion of the gene encoding CypD, a mitochondrial protein in MAMs, reduced the abundance of MAMs and impaired their integrity. Interestingly, mice lacking CypD had insulin resistance and elevated hepatic neoglucogenesis in insulin tests. Treating CypD knockout mice with Metformin significantly improved the integrity of MAMs and the insulin sensitivity (Tubbs et al., 2014; Stacchiotti et al., 2018; Figure 6). Therefore, maintaining the stability of MAMs is a necessary condition for stabilizing intracellular Ca^2+^ and increasing insulin sensitivity. This is an essential direction for treating diabetes and reducing the incidence of CVDs.

## Smoking

**FIGURE 7:**
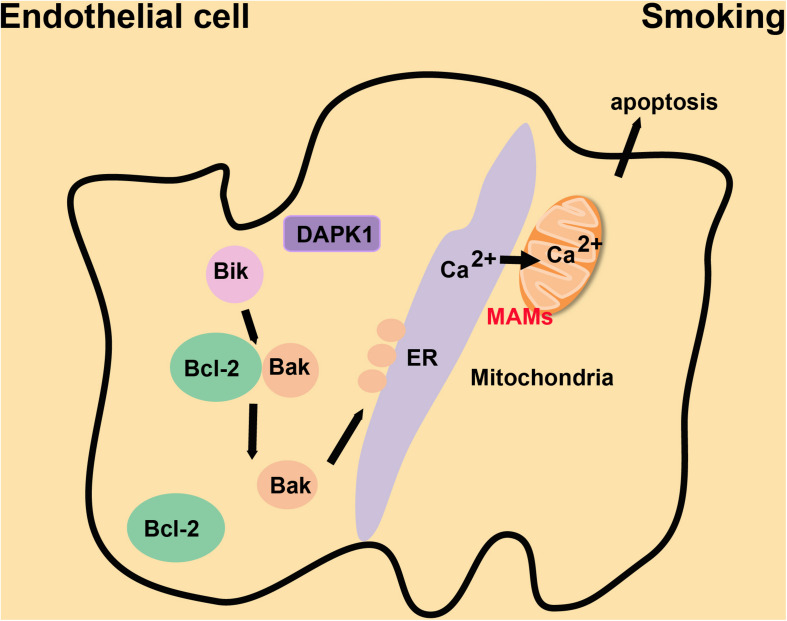
The mechanism by which MAMs repress cigarette smoke-induced proliferation of endothelial cells. Bak is enriched at the ER following Bik-dependent dissociation of the Bak/Bcl-2 complex. Bak interacts with DPAK1 on the ER to increase the abundance of MAMs and Ca^2+^ flow from the ER to mitochondria, which suppresses the proliferation of endothelial cells induced by cigarette smoke.

Smoking is one of the critical risk factors in the occurrence and development of CVDs (Desgraz et al., 2017). Long-term smoking also inhibits the proliferation and repair responses of airway epithelial cells (Wang et al., 2001). Nicotine, as a standard component of cigarette smoke, induces bronchial epithelial cell senescence and apoptosis via ROS-mediated impairment of autophagy (Bodas et al., 2016). The Ca^2+^ reservoir in the ER is decreased in chronic obstructive pulmonary disease patients, and the Ca^2+^ influx is suppressed in epithelial cells from smokers (Petit et al., 2019; Figure 7). The following mechanism, based on a study of mice and cultured cells, may explain this phenomenon. Bik (Bcl-2 interacting killer) reduces the proliferation of epithelial cells by causing the release of Ca^2+^ stored in the ER. Bik dissociates the Bak/Bcl-2 complex, leading to the enrichment of Bak around the ER. Bak interacts with the kinase domain of DAPK1, increasing the abundance of MAMs and thus the flow of Ca^2+^ from the ER to mitochondria, which causes the apoptosis of proliferating epithelial cells to reduce cigarette smoke-induced mucous cell hyperplasia (Mebratu et al., 2017; Figure 7). Inhalation of cigarette smoke results in immune system imbalances which induce exaggerated and prolonged inflammation in the lung (Racanelli et al., 2018) and contribute to the development of CVDs. Some data suggest that folic acid and Vitamin B_12_ may combat oxidative stress caused by smoking via supplying essential nutrients, removing free radicals and inhibiting inflammation (Bhattacharjee et al., 2016). However, further exploration is required to determine whether these vitamins can regulate the proliferation and apoptosis of endothelial cells by affecting enrichment of MAMs in endothelial cells. Inhalation of cigarette smoke decreases the flow of Ca^2+^ and increases the production of ROS, which inhibits the proliferation and self-recovery ability of endothelial cells, and then causes apoptosis. The same effect will occur when MAMs are enriched in endothelial cells. Therefore, exploring the functional changes of MAM structure during inhalation of cigarette smoke will be important in devising methods to lessen the inflammatory damage and reduce complications such as CVDs.

## Hypertension

In pathology, hypertension is divided into two categories: primary hypertension and secondary hypertension. Primary hypertension, the main form of hypertension, is mainly due to structural and functional changes in small and large arteries, which cause high blood pressure (Laurent and Boutouyrie, 2015). Secondary hypertension, which affects about 5–10% of the hypertensive population, refers to hypertension caused by another disease or medical condition (Rimoldi et al., 2014). It includes renal vascular hypertension, primary aldosteronism, and diabetes-related hypertension (Laurent and Boutouyrie, 2015). Vascular constriction, diminished vasodilation reserve and stenosis, eutrophic remodeling, and changes in expandability are characteristics of small resistance arteries in patients with essential hypertension (Folkow, 1982, 1995; Mulvany and Aalkjaer, 1990; Schiffrin, 1992; Heagerty and Izzard, 1995; Mulvany et al., 1996; Rizzoni and Agabiti-Rosei, 2012). Proliferation, mild inflammation, fibrosis, and chronic vasoconstriction of VSMCs are implicated in the remodeling of hypertensive arterioles (Intengan and Schiffrin, 2001; Schiffrin and Touyz, 2004). These factors also affect the formation and development of atherosclerotic plaques. VSMCs control vascular homeostasis, including dilation, contraction, and remodeling (Moulis et al., 2019).

**FIGURE 8:**
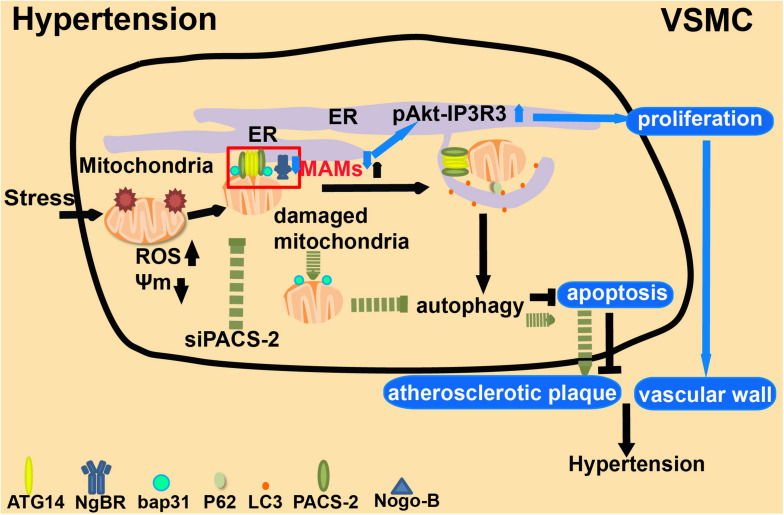
The role of MAMs in hypertension. 1. Under extracellular stresses, PACS-2-associated MAM contacts are formed in VSMCs, which mediate mitophagy (autophagic degradation of mitochondria). 2. Depletion of PACS-2 decreases both mitophagy activity and the abundance of MAMs. This induces apoptosis and stimulates the formation of atherosclerotic plaques. 3. NgBR affects cell proliferation and migration by interacting with its ligand Nogo-B. Low expression of NgBR reduces the enrichment of MAMs and promotes pAkt-IP3R3 signaling, which enhances the proliferation of VSMCs and causes clinical symptoms.

Recent studies also suggested a link between MAMs, autophagy and hypertension. PACS-2 maintains the migration of the autophagy-initiating ATG14 complex into the early autophagosome assembly region of the ER in the MAM structure (Hailey et al., 2010; Hamasaki et al., 2013). Depletion of PACS-2 induces BAP31-dependent mitochondrial fission and the dissociation of MAMs (Simmen et al., 2005). During stress, MAM structures will accumulate in VSMCs, and at the same time, PACS-2 will gather at MAMs. Depletion of PACS-2 will diminish mitochondrial autophagy in VSMCs and decrease the abundance of MAMs, thus inducing apoptosis (Moulis et al., 2019; Nahapetyan et al., 2019) and eventually leading to the development of hypertension (Figure 8). NgBR, localized in the ER, affects proliferation and migration by interacting with its ligand Nogo-B in VSMCs (Miao et al., 2006). NgBR is also required for angiogenesis (Zhao et al., 2010) and development (Rana et al., 2016). Its expression is low in the thickened pulmonary arteries of a hypoxic pulmonary hypertension rat model. Downregulation of NgBR expression reduces the abundance of MAMs in VSMCs, and meanwhile, promotes pAkt-IP3R3 signal transduction on the surface of MAMs, enhancing the proliferation ability of VSMCs (Yang et al., 2019b; Figure 8). Accumulating evidence indicates that MAMs may affect the functional structure of the vascular wall by regulating the proliferation, migration, and apoptosis of VSMCs, which may cause clinical symptoms such as pulmonary hypertension. Reducing the accumulation of MAMs may have therapeutic value for ameliorating the structural damage of blood vessels, and may provide new strategies for preventing arterial hypertension (Yang et al., 2019c).

## Hypercholesteremia

**FIGURE 9:**
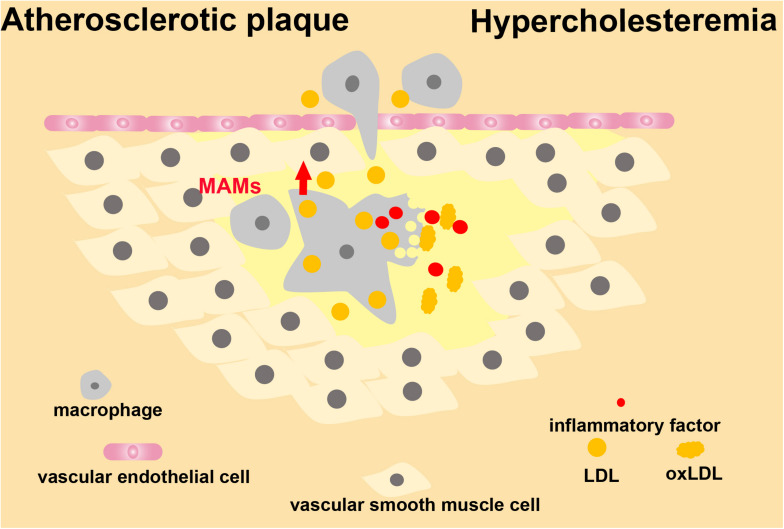
MAMs in the development of atherosclerosis. MAMs are involved in signal transduction of macrophages during hypercholesteremia-induced chronic inflammation of the vascular wall.

The lipoprotein transport system is vital to human health (Genest, 2003). Lipoproteins are classified according to size and density. HDL is relatively heavy as compared to LDL. However, chylomicrons, chylomicron residues, and VLDL are larger and lighter. Among lipoproteins, LDL particles are the main carriers of cholesterol to peripheral tissues, and loss-of-function analysis indicates that the LDL receptor is the main factor leading to hyperlipidemia (Geovanini and Libby, 2018). According to the lipid hypothesis, LDL is the key to reducing atherosclerotic plaque formation and limiting complications (Ridker, 2016). In hypercholesteremia, cholesterol-carrying LDL particles remain in the arterial wall (Williams and Tabas, 1995; Skalen et al., 2002; Hansson, 2005). This localization causes local inflammation within blood vessel walls, differentiation of monocytes into macrophages, accumulation of intracellular cholesterol, and production of inflammatory mediators (Libby et al., 2011). Consequently, immune cells are continuously recruited to secrete immune factors, leading to chronic inflammation (Stemme et al., 1995; Frostegard et al., 1999; Hansson and Hermansson, 2011). Oxidative stress stimulates vascular tissue to produce oxLDL, which is a critical trigger for atherosclerosis progression (Suciu et al., 2018). With the accumulation of oxLDL in the subendothelial region, the endothelium will become dysfunctional and undergo permeability changes (Palinski et al., 1989; Liao, 2013). At this stage, the role of MAMs in endothelial cells during the formation of atherosclerotic plaques remains unclear. However, during chronic inflammation of the vascular wall caused by hypercholesteremia, MAMs in macrophages are involved in transducing signals. Increasing evidence shows that the formation of NLRP3 inflammasomes is a key step in the process of atherosclerotic plaque formation caused by oxLDL (Lundberg and Yan, 2011; Figure 9). When inflammatory responses are triggered in macrophages, mitochondria lose their membrane potential, mtROS is upregulated, and the downstream pathways are activated to release inflammatory factors. Following the formation of inflammasomes at MAMs, NLRP3 accumulates at mitochondria, probably by sensing the increased calcium level (Triantafilou et al., 2013; Namgaladze et al., 2019). In patients with hypercholesteremia, new breakthroughs will be reached by studying the structure of MAMs in macrophages in the atherosclerotic plaque, and by understanding how MAMs regulate the development of chronic inflammation of blood vessels. We must also pay attention to the changes of MAM structures in vascular endothelial cells in atherosclerotic plaques, which may lead to new research directions.

## Conclusion

Recently, the structure and function of MAMs have been intensively investigated. MAMs are involved in many aspects of cellular activities and are essential structures to maintain cell homeostasis and mutual communications between organelles. In certain circumstances, inflammation develops when the structure and function of MAMs are disturbed in some specialized types of cells, thus aggravating the progression of CVDs. Smoking, obesity, hyperglycemia, hypertension, hyperlipidemia, and other high-risk factors of CVDs will cause alterations of MAMs in different tissues, affecting the patient’s prognosis. However, the growing body of recent experimental evidence mainly focuses on macrophages. Studies on vascular smooth muscle and endothelial cells are scarce, especially in the process of atherosclerosis. In the future, we need to explore the role of structural and functional changes in MAMs, and specific therapeutic strategies in smooth muscle and endothelial cells. MAMS are important places for mitochondrial fission, autophagy, intracellular energy metabolism, and material exchange; therefore, it is possible that controlling the structural changes of MAMs may be a key to treating patients with CVDs.

## Author Contributions

DF and JL conceived the review. HL, HF, and DF wrote the manuscript with the input from HZ, XL, DZ, YX, and PH. XL and HF drew the cartoons. All authors discussed the manuscript.

## Conflict of Interest

The authors declare that the research was conducted in the absence of any commercial or financial relationships that could be construed as a potential conflict of interest.

## Abbreviations

AMI: acute myocardial infarction
AMPK: AMP-activated protein kinase
ASC: apoptosis-related speck-like protein
Bik: Bcl-2 interacting killer
BNIP3: BCL2/adenovirus E1B interacting protein 3
CAD: coronary artery disease
CaMKII δ: Ca2^+^/calmodulin regulated kinase δ
CVD: cardiovascular disease
CypD: cyclophilin D
DCM: dilated cardiomyopathy
FUNDC1: FUN14 domain containing 1
GSK3b: glycogen synthase kinase 3b
Hcy: homocysteine
HDL: high-density lipoprotein
HF: heart failure
HHcy: hyperhomocysteinemia
I/R injury: ischemia-reperfusion
IFN- γ: interferon γ
IP3R2: inositol 1,4,5-trisphosphate type 2 receptor
IRGM: immunity-related GTPase M
LDL: low-density lipoprotein
MAMs: mitochondrial-associated membranes
mtDNA: mitochondrial DNA
mt-ROS: mitochondrial reactive oxygen species
NgBR: Nogo-B receptor
NLRP3: NOD-like receptor family pyrin domain-containing protein 3
oxLDL: oxidative low-density lipoprotein
PACS-2: phosphofurin acidic cluster sorting protein 2
RyRs: ryanodine receptors
SR: sarcoplasmic reticulum
T2D: type 2 diabetes
Tet2: ten-eleven translocation 2
VDAC1: voltage-dependent anion-selective channel protein 1
VSMCs: vascular smooth muscle cells.
